# Hemobilia and hemocholecyst as an unusual presentation of gallblader cancer: Report of a case

**DOI:** 10.1016/j.ijscr.2022.107712

**Published:** 2022-09-28

**Authors:** Deysi Laura Navarrete Espinosa, Carlos de Jesús Cocom Quijano, Carlos Daniel Torres García, Rashid Israel Arjona Bojórquez, Otoniel Escobar Flores, Orson Raziel Juan Hernández, Aron Cervantes Sánchez, Jonathan Esaú Ceseña Barrientos

**Affiliations:** General Surgery Department, Hospital General Regional 1 “Ignacio García Téllez”, IMSS, Mérida, Yucatán. Zip Code: 97150, Mexico

**Keywords:** GC, gallbladder cancer, BDE, bile duct exploration, CBD, common bile duct, RUQ, right upper quadrant, GI, gastrointestinal, HCC, hepatocellular carcinoma, US, ultrasonography, CT, computed tomography, MR, magnetic resonance, Hemobilia, Hemocholecyst, Gallbladder, Adenocarcinoma, Biliary tract

## Abstract

• The most common causes of hemobilia are iatrogenic, trauma related and neoplastic.
• The most common spontaneous cause of hemobilia is primary or metastatic hepatobiliary malignancy.
• Hemobilia is a (1–7 %) rare presenting sign of biliary neoplasm.
• Hemobilia's classic presentation triad is right upper quadrant (RUQ) pain, GI bleeding, and jaundice.

## Abstract (es)

**Introduction and importance:**

Gallbladder cancer (GC) manifesting as bilioma and hemocholecyst is rare. There are few cases reported in the literature to date. We present a case of gallbladder cancer diagnosed after cholecystectomy with emergency bile duct exploration (BDE).

**Case presentation:**

A 71-year-old female patient is admitted to the emergency department with the presence of abdominal pain with a diagnosis of acute cholecystitis. Cholecystectomy was performed with BDE, finding blood and clots inside the gallbladder and common bile duct (CBD) Intraoperatively, a tumor is identified in the fundus of the gallbladder. The definitive histopathological report reports poorly differentiated adenocarcinoma of the gallbladder.

**Clinical discussion:**

The most common spontaneous cause of hemobilia is primary or metastatic hepatobiliary malignancy. Diagnosis of hemobilia can be challenging because of its uncommon occurrence, especially in instances where there is no history of biliary tract manipulation or trauma. In cases where there is no high index of suspicion, hemobilia is often recognized late.

**Conclusion:**

It is important to take into account among the differential diagnoses of acute cholecystitis gallbladder cancer manifested as hemobilia that can cause obstruction of the biliary tract and abdominal pain.

## 1 Introduction

Hemobilia refers to the extravasation of blood into the biliary tract. The most common causes of hemobilia are iatrogenic, trauma related and neoplastic [1].

Gallbladder adenocarcinoma is a rare gastrointestinal malignancy and is the most common histologic subtype, making up 76 % of all gallbladder neoplasms. These malignancies are most often discovered incidentally, in 1 % of all cholecystectomy procedures and it is associated with a poor prognosis, with a mean survival of 6 months and a 5 year survival of 5–20 %.In the United States, the incidence of gallbladder adenocarcinoma is one to two cases per 100,000 people. It is more prevalent in women than men. Risk factors include cholelithiasis, porcelain gallbladder, gallbladder polyps, primary sclerosing cholangitis, chronic infection, congenital biliary cysts, abnormal pancreaticobiliary duct junction, obesity and medications.

Hemobilia is a (1–7 %) rare presenting sign of biliary neoplasm [2]. Though hemobilia remains an uncommon cause of digestive tract bleeding, its incidence has gradually increased as the arsenal of minimally invasive hepatopancreatobiliary procedures has expanded [3]. Hemobilia's classic presentation triad is RUQ pain, Gastrointestinal (GI) bleeding, and jaundice. Diagnosis of hemobilia can be challenging because of its uncommon occurrence, especially in instances where there is no history of biliary tract manipulation or trauma. In cases where there is no high index of suspicion, hemobilia is often recognized late [4].

This patient was managed in a public healthcare system setting. This case report has been reported in line with the SCARE 2020 criteria [5].

## 2 Presentation of a case

A 71-year-old female patient was admitted to the emergency department in a wheelchair with her family member, referred by her family doctor due to the presence of intense abdominal pain in the epigastrium, nausea and vomiting. On admission, a drowsy patient, with uncontrolled blood pressure, scleral jaundice, tachycardia and intense abdominal pain in the epigastrium, it is important to mention that the patient never presented lower gastrointestinal bleeding. Her past medical and surgical history included obesity, diabetes and long-standing arterial hypertension treated with insulin and nifedipine, chronic kidney disease on peritoneal dialysis for a year.

An ultrasound is performed that reports an enlarged gallbladder of 113 × 35 × 39 mm, with the presence of a 13-mm stone inside the neck and echogenic material suggestive of acute cholecystitis (the ultrasound image is not available, only the written report); laboratory tests revealed a white blood cell (WBC) count of 17,000/μL and biliary tract obstruction with total bilirrubin (TB) 3.8 mg/dL, direct bilirrubin (DB) 2.63 mg/dL, indirect bilirrubin (IB) 0.75 mg/dL, alkaline phosphatase (ALP) 639 U/L, alanine transaminase (ALT) 185 U/L, aspartate transaminase (AST) 311 U/L, gamma-glutamyl transferase (GGT) 425 U/L. There were signs of peritoneal irritation and patient was unstable.

**Image 1:**
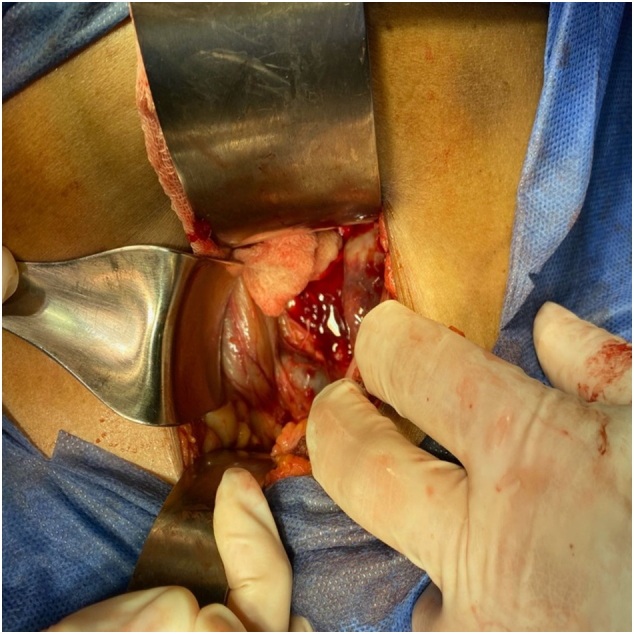
Right subcostal wound where hemocholecyst is visualized.

**Image 2:**
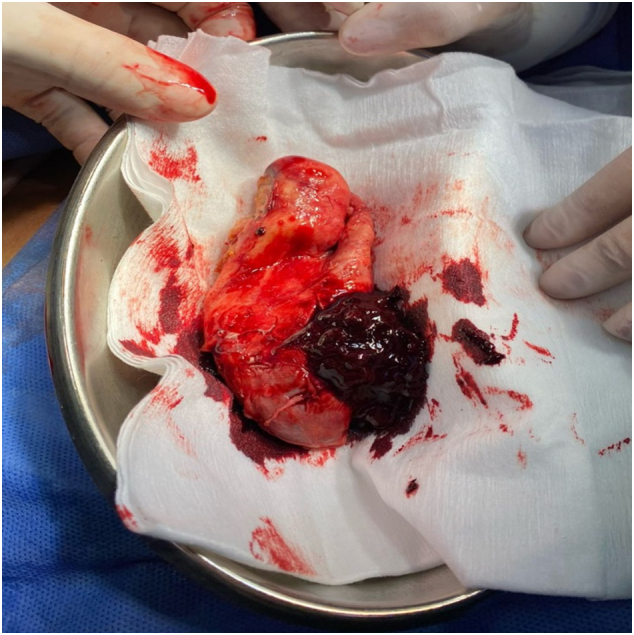
Gallbladder with abundant clots in its interior.

**Image 3:**
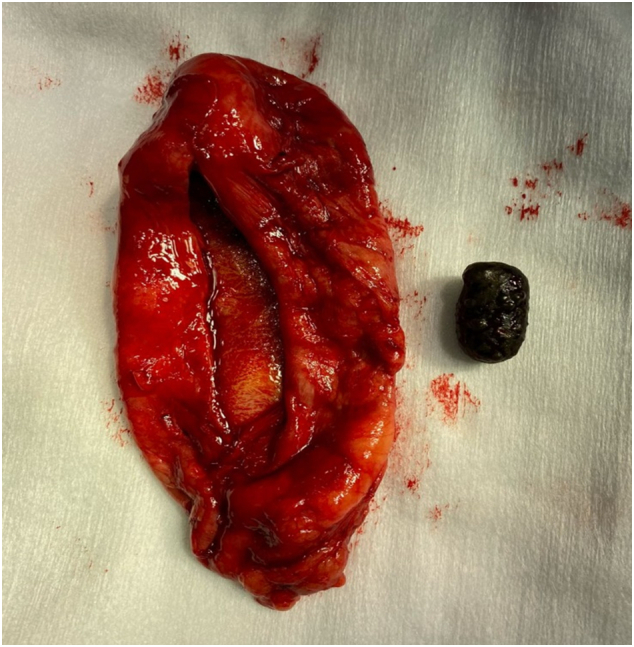
Single stone found in the vesicular neck.

**Image 4:**
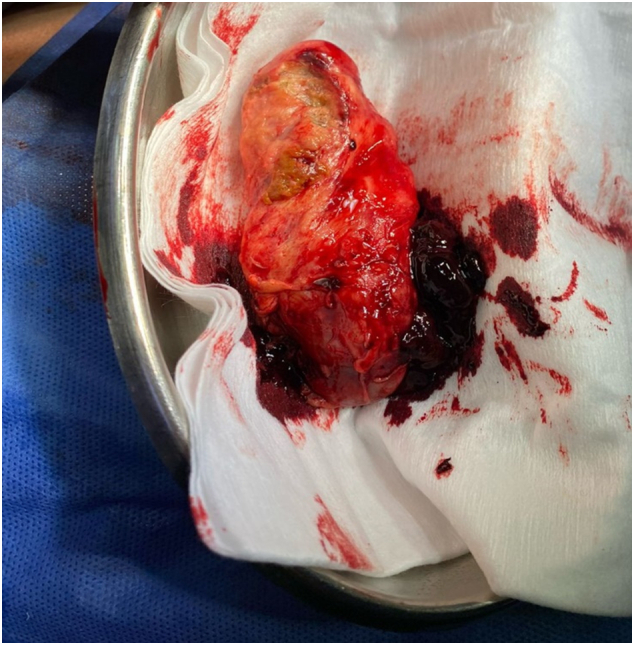
Tumor is visualized in the vesicular fund.

It is decided to perform an emergency open cholecystectomy, which is performed by the general surgeon in charge of the patient. During surgery there are abundant clots and a stone inside the gallbladder (Image 1, Image 2, Image 3) a tumor is found in the gallbladder fundus (Image 4), it is decided to explore the CBD due to the presence of dilation of approximately 12 mm. Bile duct exploration was performed by choledochotomy and instrumentation with randalls fórceps finding abundant clots in CBD without stones inside.

On the follow-up, WBC and liver function tests were within the normal range. During her post-surgical stay, extension studies were requested, however the patient requested her voluntary discharge without accepting more treatments or interventions. The pathology report determined a tumor in the gallbladder fundus of 3 × 2 × 2 cm with the type and histological grade of poorly differentiated adenocarcinoma of the gallbladder without the presence of extension to the liver, it was classified as pT2b pNx pMx.

The final outcome was the death of patient at 2 months.

## 3 Discussion

The first reported case of hemobilia dates to 1654, when Francis Glisson wrote of a nobleman whom, while dueling, suffered a blow to the right upper abdomen leading to massive gastrointestinal bleeding and ultimately death. Literature from the early 1900s began to contain various case reports of biliary tract bleeding, but it was notuntil 1948 that the specific term ‘hemobilia’ was coined [1]. The presence of blood inside the gallbladder can occur for several reasons: acute cholecystitis, abdominal trauma, liver biopsy, biliary neoplasms, hemobilia, heterotopic gastrointestinal mucosa, aneurysms of arteries neighboring the gallbladder, hemorrhagic diathesis, biliary parasitosis and ischemia [6].

The most common spontaneous cause of hemobilia is primary or metastatic hepatobiliary malignancy. This is thought to be due to more friable tissue and vasculature, leading to an increased propensity for spontaneous hemorrhage. Hepatobiliary malignancies, including cholangiocarcinoma, pancreatic cancer, gallbladder cancer, liver metastasis, and hepatocellular carcinoma (HCC) have all been associated with hemobilia. All cause malignancies account for 10 % of total hemobilia cases [3] Hemocholecyst occurs in less than 1 % of gallbladder tumors and manifests with nonspecific symptoms, such as abdominal pain in the RUQ and fever [6].

Many authors have different presentations of GC, including empyema, cholecystitis, benign biliary stricture, liver abscess, gastric outlet obstruction, and carcinoma of the head of the páncreas [7].

The classic presentation of hemobilia is described by Quinke's triad: RUQ pain, jaundice and gastrointestinal bleeding, with all three present in only 25–33 % of cases [8]. Our patient presented abdominal pain as main symptoms, later in the hospital physical examination documented jaundice of the sclera and the laboratory documented obstruction of the biliary tract, she never presented digestive tract bleeding because the gallbladder and CBD were full of clots.

The best imaging modalities for diagnosing these tumors are ultrasonography (US) and computed tomography (CT) scans [7]. US is the most frequently performed initial test in acute biliary pathology [8] and has been used for a long time in the diagnosis of acute cholecystitis. Unfortunately, polyps and carcinomas have echogenicity similar to the gallbladder wall, making it difficult to distinguish them from a thickened wall secondary to acute inflammatory changes [7]; its content can be non-shadowing or fluid levels caused by the blood components or accumulations of clots, which can present as clumps of echoes [9].

US has the advantage of availability at emergency departments, portability, the absence of ionizing radiation, and its high sensitivity and specificity for biliary pathology. However, it has limitations, such as the patient habitus, uncooperative patients, and being operator dependent. [9] Endoscopic ultrasound can assist in distinguishing benign from malignant disease, but given its more invasive nature, it is not used routinely [7].

CT carries high sensitivity and specificity in the identification of a gallbladder mass, but lower sensitivity in identifying nodal spread, and is therefore not considered ideal for staging. Angiography has also been used in some centers with questionable efficacy. More recently, magnetic resonance (MR) and cholangiopancreatography has evolved into a very sensitive and specific technique that can also help in the staging process. However, in the presence of a hemocholecyst all these imaging modalities become less sensitive. Given the rarity of gallbladder cancer, and the even rarer association with a hemocholecyst, prompt diagnosis still eludes the average radiologist, surgeon, and clinician [7]. In our patient, only an ultrasound was performed, which documented an enlarged gallbladder with thick walls and a stone in the neck that suggested acute cholecystitis. The suspicion of clots or the presence of the gallbladder fundus tumor that was found during surgery was not documented.

Complete resection of the tumor provides the only chance of cure, but an early preoperative diagnosis is essential. In fact, the 5-year survival rate after resection with curative intent has been reported at less than 17 %. [6] In our case, the diagnostic suspicion of gallbladder cancer was carried out intraoperatively when a tumor was found in the gallbladder fundus after complete cholecystectomy. It was confirmed later with the histopathological study.

## 4 Conclusion

Hemobilia and hemocholecyst secondary to gallbladder cancer are infrequent, however, they should be considered in the differential diagnosis of pain in the right hypochondrium and symptoms of biliary tract obstruction to evaluate a complete preoperative surgical plan.

## Funding

This work has no funding sources.

## Ethical approval

This manuscript is a case report retrospectively and also is not a clinical study. The ethical approval is not necessary. the institution is exempt from ethical approval.

## Provenance and peer review

Not commissioned, externally peer-reviewed.

## Consent

Written informed consent was obtained from the patient for publication of this case report and accompanying images. A copy of the written consent is available for review by the Editor-in-Chief of this journal on request.

## Author contribution

Writing the paper & assisting in the procedure: Deysi Laura Navarrete Espinosa; Carlos de Jesús Cocom Quijano; Carlos Daniel Torres García.

Performing the procedure & assisted in Literature search: Deysi Laura Navarrete Espinosa; Orson Raziel Juan Hernández; Aron cervantes Sánchez; Otoniel Escobar Flores.

Assisted in writing manuscript: Deysi Laura Navarrete Espinosa; Carlos de Jesús Cocom Quijano.

Data/evidence collection: Carlos Daniel Torres García; Otoniel Escobar Flores; Jonathan Esaú Ceseña Barrientos.

Designed the study: Deysi Laura Navarrete Espinosa; Carlos de Jesus Cocom Quijano.

Review of the manuscript: Carlos Daniel Torres García; Rashid Israel Arjona Bojórquez.

Literature Review and guarantor: Carlos de Jesús Cocom Quijano; Deysi Laura Navarrete Espinosa.

## Guarantor

Carlos de Jesús Cocom Quijano; Deysi Laura Navarrete Espinosa.

## Registration of research studies

Does not apply.

## Declaration of competing interest

There are no conflicts of interests.
